# Perspective: Plant-Based Meat Alternatives Can Help Facilitate and Maintain a Lower Animal to Plant Protein Intake Ratio

**DOI:** 10.1016/j.advnut.2023.03.003

**Published:** 2023-03-10

**Authors:** Mark Messina, Alison M. Duncan, Andrea J. Glenn, Francois Mariotti

**Affiliations:** 1 Soy Nutrition Institute Global, Washington, DC, USA; 2 Department of Human Health and Nutritional Sciences, University of Guelp, Guelph, Ontario, Canada; 3 Department of Nutrition, Harvard T.H. Chan School of Public Health, Boston, MA, USA; 4 Department of Nutritional Sciences, University of Toronto, Toronto, Ontario, Canada; 5 Toronto 3D Knowledge Synthesis and Clinical Trials Unit, Clinical Nutrition and Risk Factor Modification Centre, St. Michael’s Hospital, Toronto, Ontario, Canada; 6 Université Paris-Saclay, AgroParisTech, INRAE, UMR PNCA, Palaiseau, France

**Keywords:** plant-based meat alternatives, animal protein, plant protein, legumes, sustainability, nutrient fortification, shortfall nutrients

## Abstract

The health and environmental advantages of plant-predominant diets will likely lead to increasing numbers of consumers reducing their reliance on animal products. Consequently, health organizations and professionals will need to provide guidance on how best to make this change. In many developed countries, nearly twice as much protein is derived from animal versus plant sources. Potential benefits could result from consuming a higher share of plant protein. Advice to consume equal amounts from each source is more likely to be embraced than advice to eschew all or most animal products. However, much of the plant protein currently consumed comes from refined grains, which is unlikely to provide the benefits associated with plant-predominant diets. In contrast, legumes provide ample amounts of protein as well other components such as fiber, resistant starch, and polyphenolics, which are collectively thought to exert health benefits. But despite their many accolades and endorsement by the nutrition community, legumes make a negligible contribution to global protein intake, especially in developed countries. Furthermore, evidence suggests the consumption of cooked legumes will not substantially increase over the next several decades. We argue here that plant-based meat alternatives (PBMAs) made from legumes are a viable alternative, or a complement, to consuming legumes in the traditional manner. These products may be accepted by meat eaters because they can emulate the orosensory properties and functionality of the foods they are intended to replace. PBMAs can be both transition foods and maintenance foods in that they can facilitate the transition to a plant-predominant diet and make it easier to maintain. PBMAs also have a distinct advantage of being able to be fortified with shortfall nutrients in plant-predominant diets. Whether existing PBMAs provide similar health benefits as whole legumes, or can be formulated to do so, remains to be established.

**Statement of Significance**

To our knowledge, the submitted manuscript is the first to weigh the advantages and disadvantages of using plant-based meat alternatives (PBMAs) to lower the dietary animal to plant protein intake ratio. It compares the advantages of PBMAs with meat and with legumes consumed in the traditional manner.

## Introduction

For the past several decades, health organizations have recommended increasing the intake of whole plant foods [1]. These recommendations include consuming more fruits and vegetables [2,3], whole grains [4], and fiber-rich foods [5]. To varying degrees, following this dietary advice is associated with reduced risks of cancer, cardiovascular disease, diabetes, and overall mortality [[6], [7], [8], [9]]. More recently, the health benefits of replacing animal protein with plant protein have been emphasized [[10], [11], [12], [13]]. The environmental advantages of this exchange are also highlighted [[14], [15], [16]].

In developed countries, considerably more protein is consumed from animal sources than from plants. For example, NHANES (2015–2018) data indicate the US animal to plant protein intake ratio is approximately 2.1:1 (54.8 g/d animal protein, 25.8 g/d plant protein) [17]. Similar ratios have been reported in many developed countries [18,19].

Although plant protein composition (i.e., amino acids) may play a role [13,20], the health benefits associated with consuming diets higher in plant protein likely rather result, at least partly, from the nonprotein components of whole food sources of plant protein. It is therefore noteworthy that in the United States, 46% of the plant protein intake is from refined grains [21]. Similarly, in France, one-third of dietary protein intake is from plant sources and of that, 55% is from refined grains [18]. As noted by Perraud et al. [22] and Mariotti and Huneau [23], protein intake profiles largely define diets because protein food sources contribute other nutrients that tend to cluster together as a “protein package”, e.g., red meat tends to contribute saturated fatty acids and bioavailable iron, and foods high in plant protein tend to contribute fiber and polyunsaturated fatty acids.

The optimal dietary intake ratio of animal to plant protein has not been established and may differ according to food availability. The EAT–Lancet Commission recommends obtaining *most* protein from plant sources [14], whereas Canada’s Food Guide states “Choose protein foods that come from plants more often” when referring to “protein foods” [24], and the Health Council of the Netherlands recommends a 1:1 animal to plant protein ratio [25]. The latter 2 recommendations align with efforts to reduce, but not eliminate, the dietary intake of animal products, especially meat [26,27]. These recommendations can still result in a marked reduction in diet-related environmental pressures, such as reducing greenhouse gas emissions, and land and water use [22] and are more likely to succeed because relatively few people will ever completely eliminate animal protein from their diet [[28], [29], [30]], whereas many may be willing to substantially reduce their intake [31,32].

Effective reduction in meat consumption (flexitarianism) or intention to do so (proflexitarianism) is largely explained by current attitudinal changes related to the impact of meat intake on human health, climate change, and animal welfare [33]. However, meat has an important culinary and cultural role in most societies [34,35] and is viewed as an essential part of a meal in many cultures [36,37]. Consequently, even complying with the Dutch recommendation to consume similar amounts of protein from animal and plant sources will be a challenge for populations in developed countries, despite there being many readily available plant sources of protein.

The premise of this paper is that the new generation of plant-based meat alternatives (PBMAs) can play a pivotal role in this protein intake transition from animal to plant protein sources. Not only can PBMAs aid in the transition to a diet higher in plant protein, they can also help to maintain that diet once the transition has been made. The role of PBMAs as foods facilitating the transition from an animal-based to a more plant-predominant diet is not a new concept [[38], [39], [40]]. Less appreciated is the utility of PBMAs in maintaining that newly transformed plant-predominant diet over the long term [41]. Maintenance of a plant-predominant diet is an important consideration because a US study found that 10% of adults (≥17 y) are former vegetarians/vegans whereas only 2% are currently vegetarian or vegan [42]. Thus, ensuring continued adherence to a plant-predominant diet may be a seminal advantage of PBMAs. Newly developed PBMAs are designed to provide the consumer with the same orosensory properties as and functionality of the meat products they are intended to replace. For these reasons, these products are more likely to be embraced by populations in developed countries where meat has come to play a central role in the culture [43].

Given the potential role of PBMAs in the transition to and maintenance of a diet that contains more plant protein, it is important to consider their nutritional and health attributes in comparison to both the meat they replace and the whole plant foods (legumes, grains, nuts, seeds) for which they serve as an alternative [16,44,45]. Globally, dietary guidelines provide relatively little counsel on the use of PBMAs [46]. An exception is Canada’s Food Guide, which notes in their guidance on how to eat more protein foods that come from plants, that many simulated meat products are highly processed and can add excess sodium and saturated fat to the diet, and therefore, it is important to use food labels to make a healthy choice [47].

Thus, the ramifications of reducing meat consumption on nutrient intake must be considered because meat contributes significantly to nutrient intake globally [48], including in Europe [49] and the United States [50]. However, there is limited evidence of the effect of reducing meat intake on nutrient adequacy in the context of the overall diet. That is, the impact on nutrient status when meat is replaced by other specific sources of calories has not been explored. Recently, Dussiot et al. [51] ran gradual meat intake reduction scenarios in successive 10% steps for diets that were both nutritionally adequate and as close as possible to a reference healthy dietary pattern. They found that some food groups were able to replace the contribution of meat to nutrient adequacy while also leading to overall healthier dietary patterns (i.e., whole grains, fruit, vegetables, seafood). However, these healthy dietary patterns require profound reorganization of the diet.

Vieux et al. [52] estimated that approximately half of total protein intake by French adults must be animal-based to meet nonprotein nutrient-based recommendations, which would be consistent with the 1:1 dietary animal:plant intake ratio promoted by the Health Council of the Netherlands. However, the optimization model and the research strategy that the authors used have been strongly criticized, and the results appeared to be explained by a marked over-restriction of the possible solutions [53]. Also, this estimate excluded the use of dietary supplements or fortified foods and did not consider the types of more novel foods that may be consumed when meat intake is consciously reduced. Furthermore, Fouillet et al. [54] concluded that among French adults, only when plant protein accounts for more than 80% of total protein are nutrient fortification/supplementation and/or new foods required to meet nutrient requirements. In any case, all modeled diets greatly departed from current observed diets, which again indicates the major change needed to most diets.

Finally, it is acknowledged that in the near term the dietary contribution of PBMAs will be a consideration primarily relevant only to developed countries due to their high meat intake and ability to afford the relatively high cost of many PBMAs relative to more conventional meat alternatives such as legumes [55]. However, projections that the global demand for animal products will rise by 60–70% by 2050 stem primarily from the anticipated increased consumption in low- and middle-income countries [56]. As the price of PBMAs decreases, as is expected, and consumer awareness of the link between diet and climate change and the relatively low environmental footprint of PBMAs increases, these products may be embraced in both developed and developing countries [[57], [58], [59]].

The general topics covered in this manuscript include background information on PBMAs, current animal to plant protein intake ratios, protein and chronic disease risk, protein quality and global protein needs, using legumes and PBMAs to lower the animal to plant protein ratio, and current understanding of the health effects of PBMAs.

## Historical Perspective on PBMAs

Meat substitutes have existed in the Western world for more than a century and considerably longer in some Asian countries. Notable in this regard is the creation of Nuttose in 1896 by Dr. John Harvey Kellogg, which was a canned product made primarily from peanuts [60]. In 1911, in France, cold cuts made from soy became available, and in 1922, the first soy-based meat alternative was developed by Madison Foods in Tennessee [61]. However, in part because of improvements in technology and increased consumer demand, within the past 2 decades, the PBMA industry has been especially active and innovative [62].

Traditionally, adherents of plant-predominant diets met their protein needs by consuming a mix of grains and legumes, a common culinary practice among cultures throughout the world. These food groups have indispensable amino acid (IAA) profiles that complement one another such that the combination produces a protein with an IAA pattern that more closely matches biological requirements [[63], [64], [65], [66]]. Traditional soy foods such as tofu and tempeh, which have been consumed in many parts of Asia for centuries, have been embraced by flexitarians [67] and vegetarians over the past several decades because of the abundant amounts of high-quality protein they provide [68,69]. There are also “veggie” burgers, such as a black bean burger, made using beans, often in combination with grains and vegetables that may or may not be vegan [70]. This type of product is especially attractive to those wanting a food that can be consumed in the same manner as a meat patty. The wheat (gluten)-derived product seitan also fills a role as a meat substitute [71].

However, none of the aforementioned products truly emulate the orosensory properties of meat, and for this reason, their entrance and acceptance by the mainstream consumer has been somewhat limited [72,73]. Research shows that for PBMAs to successfully replace meat, they need to emulate the taste, texture, visual appearance, and cooking method of meat [74,75]. The new generation of PBMAs, which are typically comprised of protein extracted from legumes such as pea and soy, and/or wheat gluten and require new technology for their production, meets these criteria [45,76,77]. These products, while embraced by vegetarians, are designed to appeal to a much broader demographic—basically, anyone wanting to reduce meat intake [78]. In fact, US household data indicate that over a recent 2-y period, 86% of purchasers of PBMAs were consumers of beef [79].

Several research groups have published comprehensive reviews on the nutrient content of a wide range of PBMAs [76,[80], [81], [82], [83]], so this information will not be discussed in detail here. The nutrient content of PBMAs varies widely and differs according to the primary sources of protein, secondary ingredients (e.g., type of fat added), and the addition of micronutrients. It is also recognized that meat alternatives not made from legumes, but from algae, fungi, and insects, are gaining relevance, and that animal protein is being produced by in vitro methods and precision fermentation [84]. However, the focus of this manuscript is on legume-based PBMAs, that is, products such as patties that are made primarily from extracts of legume protein. Surveys to date indicate consumers are more accepting of PBMAs than other types of alternatives [85,86]. It is notable that a recent analysis of the French diet found that plant-based substitutes that include legumes appear more nutritionally adequate to substitute for animal products than other alternatives [87].

## Animal to Plant Protein Intake Ratios

As highlighted above, in many developed countries, approximately twice as much protein is consumed from animal sources compared with plant sources [18,19,21,88], whereas globally, this ratio is reversed—in many developing countries, much less than one-third of dietary protein is derived from animal sources [89,90]. In Bangladesh for example, 80% of the protein consumed is from plant sources [90]. Interestingly, according to crude estimates based on disappearance data, animal and plant sources provided similar amounts of protein in the United States in 1909 [91]. However, over the past century, the animal to plant protein intake ratio has steadily increased as a result of an increase in animal protein intake and a decrease in plant protein intake [21,91].

## Differences between Animal and Plant Protein

### Chronic disease risk

As noted previously, most evidence suggests that the health benefits of plant-predominant diets likely stem from the nonprotein components rather than the protein per se [92,93], and likely also from the parallel reduction in animal product intake. To this point, eliminating red and processed meat from the diet in and of itself may reduce risk of CAD [94], although there is disagreement on this point [95]. In any event, that which replaces meat in the diet likely determines the extent to which risk is reduced. For example, data from the combined analysis of the Nurses’ Health Study (*n =* 84,628 women) and the Health Professionals Follow-up Study (*n =* 42,908 men) showed that replacing 5% of energy intake from saturated fats with equivalent energy intake from polyunsaturated fat, monounsaturated fat, or carbohydrates from whole grains was associated with a 25%, 15%, and 9% lower risk of CAD, respectively, whereas replacement with carbohydrates from refined starches/added sugars was not significantly associated with risk reduction [96,97].

However, it is also possible that differences between animal and plant proteins may affect chronic disease risk because of the differences in their amino acid profiles [13,[98], [99], [100]]. For example, there is mounting evidence that the concentration of certain amino acids that is higher in animal protein than plant protein, such as the branched-chain amino acids and methionine, may exert adverse effects on metabolism, whereas the concentration of certain amino acids that is higher in plant protein than animal protein, such as glutamine, cysteine and arginine, may exert beneficial effects [20,100,101]. Thus, diets differing in the amounts of animal and plant protein content may exert differing metabolic effects due to the differences in amino acid profiles as well as the differences in nutrients and nonnutrients found in their respective protein packages. For example, there are phytochemicals such as isoflavones and glucosinolates, which theoretically could exert both favorable and unfavorable effects, and phytate, which inhibits mineral absorption but is also an antioxidant [102,103]. In general, over the past several years, systematic reviews and meta-analyses of observational data have found that animal protein is associated with increased risks of type 2 diabetes, cardiovascular mortality, and all-cause mortality, whereas plant protein is associated with decreases risks of these outcomes [12,104,105].

Finally, it would be remiss not to mention that there are shortfall nutrients in vegetarian and especially vegan diets. Notable in this regard are vitamin B12, zinc, iodine, and calcium [106]. Vegan diets require the use of supplements and/or fortified foods. However, deficiencies are much less likely to occur when adhering to a plant-predominant diet as is discussed here than a vegan diet. Furthermore, deficiencies are not necessarily more likely to occur because a serving of legumes is replaced by a PBMA. In fact, as noted later, because PBMAs have the potential to be fortified, they may be less likely to occur.

### Protein quality

Differences between the amino acid profiles of animal and plant protein are associated with differences in protein quality. Animal proteins tend to be higher in IAAs [99,107] and as such have higher protein quality scores as determined by the protein digestibility corrected amino acid score and the digestible indispensable amino acid score [[108], [109], [110]]. However, in populations consuming a mix of animal and plant protein, these differences are not likely to be clinically relevant. In fact, it is not established whether the lower quality of some plant proteins necessitates that adherents of plant-predominant diets consume higher amounts of protein to meet their biological requirements for nitrogen and amino acids [111]. To this point, there is no vegan US protein RDA as there is for iron (1.8 versus 1.0 mg). On the other hand, some [[111], [112], [113], [114]], but not all [115], experts have called for vegans to consume more protein to account for the lower digestibility of protein from plants [116]. The Dutch Ministry of Health recommends that vegans consume 30% more protein than omnivores to compensate for the lower quality of plant protein [117]; however, this recommendation greatly exceeds recommendations made by most experts [29].

### Global Protein Needs

The world population is predicted to reach close to 10 billion people 3 decades from now [118]. Determining how best to meet the global protein and caloric needs of the 2050 population is a matter of some urgency. Protein is by far the more important consideration because, as was noted in a recent report from the Stockholm Resilience Centre, “Meeting the demand for protein, within environmental limits, is one of the biggest challenges for the global food system in the 21st century” [119].

Precisely how much protein will be needed is unclear, but to answer this question, Irish researchers proposed 5 scenarios [120]. At the high end of the spectrum, it was estimated that protein production will need to increase by 78% to meet global protein needs based on the premise that the entire population will consume ∼100 g/d protein. On the low end, which is based on the premise the population will consume only 50 g/d protein, production could decrease by 13% without compromising protein availability. The high estimate may be the more probable scenario because although the types of protein consumed vary markedly among cultures and societies, if availability allows, populations tend to consume approximately 16% of their calories in the form of protein [121], which is much greater than the approximate 10% of calories needed to meet the RDA [122].

Finally, for an assortment of reasons, including concern over the environment and animal welfare as well as personal health considerations, evidence suggests that over the next 30 y in developed countries, there is likely to be a shift in the direction of a plant-predominant diet [123]. Therefore, the question becomes how the world is going to produce the calories and *plant* protein needed by 2050. If the plant protein is to come from non-soy legumes, global production would need to substantially increase to meet demand whereas worldwide soybean production is sufficient to meet that need if some of the soy protein currently used for animal feed is instead used for direct human consumption [124].

### Shifting the Animal to Plant Protein Intake Ratio

Consuming more legumes is an obvious choice for increasing plant protein intake [125, 126]. In 2013, the United Nations declared 2016 as the International Year of Pulses [127], and the International Lipid Expert Panel places soy, legumes, and nuts at the top (most desirable) of the protein source pyramid for promoting cardiometabolic health [128]. Legumes (including beans and other pulses) are beneficial to long-term health [129,130], good sources of protein and fiber [52], affordable [131], and have a low environmental footprint [132]. However, despite their many accolades, pulses are vastly underutilized sources of nutrition in most regions in the world (Table 1) [133]. Worldwide, pulses account for only 6% of total protein intake.

**TABLE 1:** World and selected region pulse intake

Region	g/d	% Total intake	
		Protein	kcal
World	21	6	3
Latin America and Caribbean	34	9	4
Sub-Saharan Africa	33	12	5
South Asia	33	11	5
North Africa	19	5	2
West Asia	19	6	3
Oceania	12	2	1
North America	11	2	1
Southeast Asia	9	3	1
Europe	7	2	1
East Asia	4	1	0
Caucasus and Central Asia	1	0	0

Adapted from reference 133 with permission.

In the United States, dry bean intake accounts for only about 2% of the total protein intake and 6% of plant protein [21]. A recent French study found that even knowledge of the health benefits of pulses did not lead to greater intake of these foods [134]. Reuzé et al. [135] recently summarized motives associated with greater legume consumption among French adults. Furthermore, only very recently (2017) did French public health authorities include a specific guideline on legumes in the dietary guidelines [136,137].

Although the terms legumes, pulses, and beans are often used interchangeably, pulses are the edible seed part of the legume plant. Pulses include beans, lentils, dry peas, chickpeas, and cow peas. Legumes not only include pulses, but soybeans, peanuts, snap beans, and snap peas [138]. Soybeans and peanuts are referred to as oilseed legumes because of their high fat content. Legume consumption has traditionally been a larger part of the cuisines in countries and regions such as Mexico (refried kidney beans), India (dhal and pappadums), the Mediterranean (navy bean soup and Greek fava), and the Middle East (falafel and hummus).

As can be seen from Table 2, on a caloric basis, legumes are approximately 29% protein, which is approximately twice the percentage (13%) found in grains, and legumes on average are also higher in fiber than grains [139]. Substituting the protein from one serving of cooked beans daily for an equivalent amount of animal protein would lower the US animal to plant protein intake ratio from approximately 2.1:1 to 1.3:1. This exchange would result in an additional 8.6 g of plant protein being consumed [140]. Adding that amount to the 25.8 g/d of plant protein per capita consumed in the United States would result in a total of 34.4 g/d [17]. Subtracting 8.6 g/d from the 54.8 g/d of animal protein per capita consumed in the United States would result in a total of 46.2 g/d. Thus, the dietary animal to plant protein ratio would be 1.3:1 (46.2:34.4). On the other hand, meeting the cooked beans, peas, and lentils intake recommendation of the current US Dietary Guidelines—3 servings weekly (1.5 cups cooked)—would clearly have much less effect [141]. If the approximate 3.7 g/d protein from these plant foods replaced an equal amount of animal protein, the ratio would decrease only from 2.1:1 to about 1.7:1 (51.1:29.5). (Note that the US Department of Agriculture designates one serving of beans as one-half cup, which weighs approximately 90 g, whereas in this manuscript, 100 g is considered to be one serving based on work by Marinangeli et al [140]).

**TABLE 2:** Protein and fiber content of selected legumes (per 100 g cooked) from the USDA’s FoodData Central^1^

Legume	USDA FDC ID	Protein		Fiber
		(g)	% kcal	(g)
Soybeans	174271	18.21	42.35	6.0
Lupin	173804	15.57	53.69	2.8
Lentils	175254	9.02	31.65	7.9
Pinto beans	175200	9.01	25.20	9.0
Great Northern beans	173790	8.33	28.24	7.0
Kidney beans (red)	175242	8.67	27.31	7.4
Black beans	175237	8.86	26.85	8.7
Mung beans	175255	7.02	26.74	7.6
Peas (green)	170102	5.36	25.52	5.5
Navy beans	173794	8.23	23.51	10.5
Adzuki beans	173789	7.52	23.50	7.3
Lima beans	169316	6.81	22.15	5.3
Garbanzo beans	173799	8.86	21.61	7.6

1 U.S. Department of Agriculture, Agricultural Research Service. FoodData Central, 2019. fdc.nal.usda.gov.

### Is the Direct Consumption of Pulses Likely to Increase?

How likely is it that Americans and other Western populations will consume one serving of cooked pulses daily? Despite calls for greater public health efforts promoting pulse consumption [142,143], there is little evidence that these efforts, if undertaken, will be successful. In NHANES (2013–2014; *n*=6048), only 4% of participants consumed legumes on both days of the survey [144]. Furthermore, that figure was lower than the 5.6% of participants who consumed legumes on both days of the survey in the previous 2 y. More recently, Tao et al. [145] reported that among the 4058 participants in NHANES 2017–2018, daily legume intake averaged only 0.11 servings, which was not higher than reported in NHANES 2011–2012 (*n =* 4313, 0.12 servings/d). This downward trend in legume intake is also evident from data from the Continuing Survey for Individual Intakes (1994–1996) wherein 14% of the US adults consumed dry beans on both days of the survey [146]. In contrast, in the 2017 Beans, Lentils, Peas Survey, only 4.9% of participants consumed beans on both days of the survey [144]. Even more striking in some ways is that, based on grocery purchases, US mean annual per capita expenditure on legumes was only $4.76 during 2017–2019 [147].

In NHANES, because of their higher fat content, neither soybeans nor peanuts are included in the legume category. As seen in Table 3, one serving (2 tbsp) of peanut butter provides almost as much protein as a serving of pulses (7.27 g versus 8.62 g) although it contains considerably more kcal (∼189 versus ∼127). Furthermore, according to the Adventist Health Study 2, reported legume intake (minus soy) of male vegans was only about 65 g/d legumes, about three-quarters of a serving, although they also consumed on average 207 g/d soy foods [148].

**TABLE 3:** Energy, macronutrient and fiber content of legumes and foods made from legumes^1^

	Pulses (*N =* 29)	Tofu (*N =* 10)	Peanut butter (*N =* 3)	Peanuts (*N =* 3)	Soy-PBMAs (*N =* 5)
Energy (kcal)	127 ± 14	100.10 ± 24.86	189.00 ± 1.73	161.33 ± 0.94	198 ± 83.28
Protein (g)	8.62 ± 1.54	10.95 ± 2.53	7.27 ± 0.38	7.23 ± 0.14	18.8 ± 5.08
% kcal protein	27.1	43.8	15.4	17.9	38.0
Fat (g)	0.66 ± 0.62	5.76 ± 1.91	16.07 ± 0.31	13.8 0.24	11.2 ± 6.97
Carbohydrate (g)	22.55 ± 3.49	2.20 ± 0.98	7.24 ± 0.40	5.03 ± 0.64	8.2 ± 2.23
Fiber (g)	7.4 ± 2.0	1.07 ± 0.60	1.99 ± 0.50	2.52 ± 0.12	4.2 ± 2.04

Nutrient database number for tofus: 16160, 16281, 16159, 16277, 16276, 16212, 16211, 16426, 16427, 16213; Peanut butter: 16097, 16398, 16167 and peanuts; 16091, 16093, 16095

PBMA, plant-based meat alternative.

1 Values = mean ± standard deviation. Serving sizes: pulses, 100 g cooked tofu, 100 g raw; peanut butter, 2 tablespoons (32 g); peanuts, 1 oz (28 g); soy-PBMA (burgers, 1 patty). Sources: Values for tofu, peanut butter, peanuts from U.S. Department of Agriculture, Agricultural Research Service. FoodData Central, 2019. fdc.nal.usda.gov. All tofus except one either hard or firm. Values for pulses and soy-PBMAs come from references [133] and [145], respectively.

Legume intake is similarly low in the United Kingdom. In the Oxford component of the European Prospective Investigation into Cancer and Nutrition, when standardized for an intake of 2000 kcal, regular meat eaters (*n =* 2852) reported consuming only 26.7 g/d of pulses/legumes. Vegans (*n =* 269) consumed 68.4 g/d, but that is still less than one serving [149]. However, vegans also consumed 59.6 g/d of vegetarian protein alternatives (e.g., tofu, soymilk, soy burgers, Quorn), much higher than the 4.9 g/d consumed by regular meat eaters.

Barriers to bean consumption in Western countries include beans not being part of the traditional diet, concerns about flatulence/abdominal discomfort, lack of knowledge about preparation/cooking [150], objectionable taste and texture and high carbohydrate content [144], the perception that beans are “poor man’s food” or “poor man’s meat,” [151] and concerns about antinutrients. Considerable efforts will be needed to increase intake of whole legumes in the general population, including additional public education campaigns, changing the food environment, and more legume-based dishes being served at schools, hospitals, and restaurants. However, if protein intake from the consumption of cooked legumes served in the traditional manner is unlikely to substantially increase, a viable alternative is to consume PBMAs made from legumes. Importantly, and as noted previously, PBMAs should not be viewed as a replacement for legumes, but as a complement; that is, it is not one or the other. Both foods can be used as a means of increasing legume and plant protein intake.

## Perspectives on PBMAs

### Introduction

The potential role of meat substitutes in reducing reliance on and changing attitudes toward meat was recently demonstrated by Bianchi et al. [152]. These authors found that a 4-wk behavioral program in which British study participants were provided PBMAs along with recipes for their use and information about the benefits of eating less meat and told of success stories of people who had reduced their meat intake, resulted in a reduction in meat intake and changes in psychosocial constructs consistent with a sustained reduction in meat intake.

Evidence that PBMAs can substantially contribute to reducing the animal to plant protein intake ratio is readily apparent. According to Messina et al. [153], the protein content of 5 soy-based patties ranged from 14 to 27 g per patty, considerably more than an average serving of pulses (∼8.6 g) (Table 3). Even a conservative estimate indicates that the consumption of only 4 servings (4 patties) of soy-based patties per week or about 75 g protein, if replacing animal protein in the diet, would bring the US animal to plant protein intake ratio down to approximately 1.2:1. Arguably, the higher protein content of the PBMAs versus legumes may allow for the former to more effectively facilitate the transition to a diet containing similar amounts of animal and plant protein.

### Skepticism about the utility of PBMAs

The increased popularity of PBMAs has led to greater scrutiny of their nutritional attributes and questions about their role in the diet. For example, Toh et al. [154] recently commented that “There is a gap in our understanding of the long-term impacts of dietary patterns that characteristically feature PBMAs compared with PBDs (plant-based diets).” The PBDs in this case refer to a balanced consumption of grains, legumes, nuts, seeds, fruits, and vegetables. Fardet et al. [155, 156] maintain that, as a rule, degrading the food matrix, as occurs for example in the extraction of protein from beans, adversely impacts the healthfulness of that food, regardless of its nutrient and calorie content. Furthermore, based on associations between ultraprocessed food (UPF) intake and adverse health outcomes, Gehring et al. [157] hypothesized that a higher intake of the processed form of plant-based foods might reduce or cancel their potential health benefits.

### Concerns about the processing involved in the production of PBMAs

The processed nature of PBMAs was highlighted by Macdiarmid [158], when this author concluded that the trend toward consuming more highly processed plant-based convenience foods is a concern regarding both public health and achieving the targets of reducing greenhouse gas emissions. If based on the NOVA classification, it was reported that swapping meat for PBMAs in France would result in an increase in the percentage of energy from UPFs from 29% in observed diets to 34% on average and up to 40% according to type of substitution in modeled diets [87]. As an aside, in reference to the comment by Macdiarmid [158], although it may be true that a soy-based patty is more convenient than having to boil dry beans, it may not be more convenient than eating canned beans or ground beef. Furthermore, research indicates that plant-based burgers have a low environmental footprint [57,58,159,160].

The considerable discussion about the NOVA food classification system and the harms of consuming foods classified by NOVA as ultraprocessed shines a negative light on the entire new generation of PBMAs because they are classified by NOVA as UPFs [161,162], foods whose intake is to be discouraged [163]. However, a recent analysis found that, of the many common criticisms of UPFs, none of those examined apply to soy-based meat alternatives (or soy-based dairy alternatives), all of which were NOVA-classified as UPFs, more so than they apply to their animal-based counterparts, beef and cow’s milk, which are classified as unprocessed/minimally processed foods. The criticisms addressed were *1*) hyperpalatability, *2*) high energy density, *3*) increased energy intake rate, *4*) low satiation, *5*) low cost/high snackability, and *6*) high glycemic index (milks only) [153]. One concern not addressed by the authors of this analysis is that UPFs cause adverse changes in the microbiome [164]; however, based on the available evidence, this does not appear to be the case for PBMAs [165].

Furthermore, many PBMAs are rated highly by nutrient profiling models other than NOVA [166]. For example, Australian researchers recently found the average Health Star rating for 50 plant burgers was 3.9 (5-point scale) versus a rating of 2.9 for a meat burger [167]. A French analysis found that plant-based meat and dairy substitutes had a small effect on overall quality of the French diet and heterogeneous impacts on nutrient adequacy and security. Importantly, as mentioned previously, it was found that plant-based substitutes that include legumes appear more nutritionally adequate to substitute animal products than other alternatives such as cereal-based substitutes [87].

### Clinical data supportive of PBMAs

Evidence offering the most direct insight regarding the comparison between meat and PBMAs comes from the clinical trial Study With Appetizing Plantfood-Meat Eating Alternative Trial (SWAP-MEAT) [168]. Participants (*n =* 36) consumed about 2.5 servings daily of PBMAs or analogous meat products, providing 25% of total calories and 50% of total protein, for 8 wk each in a randomized, crossover design. Results showed that consumption of PBMAs significantly decreased circulating levels of trimethylamine oxide and low density lipoprotein cholesterol as well as body weight when compared with the meat products [168]. There were also no differences in inflammatory markers between the groups [169] nor were adverse effects observed on any of the other endpoints analyzed.

Also of relevance are the results of the SWAP-MEAT Athlete study, which involved 24 athletes [12 recreational runners and 12 resistance trainers] who were randomly assigned to 3 diets for 4 wk each in a crossover design: whole foods plant-based, plant-based with PBMAs, and an omnivorous diet [170]. At study termination, there were no differences in running outcomes for the runners or lifting outcomes for the resistance trainers, suggesting that inclusion of PBMAs in a plant-predominant diet may not impact fitness outcomes in athletes compared to a whole foods plant-predominant diet and an omnivorous diet.

Additionally, although not a direct comparison of meat and PBMAs, a recent 3-mo parallel-arm designed clinical trial compared the effects of a low-carbohydrate vegan diet with a moderate-carbohydrate vegetarian diet in 164 participants with type 2 diabetes [171]. To achieve a lower carbohydrate content in the vegan diet, the diet was higher in fat (from canola oil) and protein. Approximately 25% of total caloric intake was derived from protein, nearly all of which came from plant sources, and much of that was derived from soy-based meat alternatives. At study termination, both diets caused significant reductions in body weight, glycated hemoglobin, blood pressure, and blood lipids, with no adverse effects reported [171].

### Legumes compared with legume-based PBMAs

As noted previously, PBMAs may be regarded as transition foods or gateway foods to facilitate the conversion to a plant-predominant diet. Or, in the words of Alae-Carews et al. [172], PBMAs are an important “stepping stone” for dietary change. Implicit in this perspective is that PBMAs are more healthful than the meat they replace but perhaps less so than less processed forms of plant protein, such as legumes and whole grains. Several authors have pondered whether PBMAs derived from legumes offer similar nutritional benefits or chronic disease reductions as whole legumes [44, 45]. Van Vliet et al. [173] went a step further by stating that “The mimicking of animal foods using isolated plant proteins, fats, vitamins, and minerals likely underestimates the true nutritional complexity of whole foods in their natural state, which contain hundreds to thousands of nutrients that impact human health.”

It is true that in addition to providing nutrients and fiber, legumes contain an assortment of phenolic compounds that may contribute to their health effects [[174], [175], [176]]. Observational data indicate legume intake is associated with reductions in risk of colorectal cancer [177] and all-cause mortality [178], and clinical data suggest pulses may help to manage body weight [179] and improve glycemic control [180]. However, the extent to which bean phenolics contribute to these associations is not known.

The new generation of PBMAs have not been commercially available long enough for their intake to be examined in observational studies, and limited clinical data are available. On the other hand, the primary proteins used in these products, such as soy, and to a much lesser extent, pea, have been rigorously investigated in the same processed form (e.g., soy protein isolate and soy protein concentrate) as that used in PBMAs. For example, soy protein is a high-quality protein [110, 181], lowers blood cholesterol levels [182, 183] and promotes gains in muscle mass and strength to a similar extent as animal proteins, including whey [184]. Furthermore, one aspect of PBMAs that is cited above by van Vliet et al. [173] as a disadvantage—the use of isolated components—may actually be an advantage in comparison to consuming legumes in their unprocessed (raw) state, as the manufacturing process allows the fortification of shortfall nutrients in both plant-predominant and animal-based diets, which, if need be, can be tailored to specific populations.

For example, one can envision that in populations where intake of a particular nutrient, such as iron, is marginal, PBMAs could serve as a vehicle for fortification of that nutrient. Industry could be encouraged to fortify their products with nutrients that may be of concern when replacing animal protein with plant protein. Interestingly, in Canada, for a product to be considered a “simulated meat product” the nutritional content must be equal to that of the meat product it is intended to substitute [185]. Of relevance to this discussion is a recent study that optimized the recipe for creating a legume-based patty aimed at improving nutritional content while taking account of technological constraints and applying nutritional constraints to limit risk of overt deficiency in 12 key nutrients. Even in this case, there remained the need to fortify with vitamin B_12_, zinc, and iron [186].

Another possible advantage of PBMAs is that components of legumes that may be objectionable to some consumers such as oligosaccharides because they can cause flatulence are greatly reduced when the protein is extracted from the beans [187]. The digestibility of the extracted protein may also be greater because of the elimination of compounds that can inhibit protein digestion [108,116]. Additionally, processing can eliminate or reduce compounds traditionally classified as antinutrients, such as phytate [188]. The nutrient content of PBMAs varies widely, but some tend to be relatively high in sodium, although they do not meet the US Food and Drug Administration definition of a high-sodium food (≥400 mg/serving) [81,153,167]. Also, some are high in saturated fat (but lower than beef), a result of the attempt to emulate the orosensory properties of meat [153].

The addition of sodium can improve the taste and flavor of plant-based ingredients and mask unpleasant flavors typical in plant-based proteins, such as beany and chalky [189]. It is important to note however, that because salt is commonly added to ground beef when cooking to improve texture and taste, there may be little difference in the sodium content between PBMAs and beef as they are typically consumed [190]. Given the improvements in taste and texture of PBMAs made in recent years, it is not unreasonable to speculate that future iterations of PBMAs will be able to maintain or improve current orosensory properties while reducing sodium and saturated fat content. In fact, the newest version of one popular soy-based burger contains less saturated fat (8 g versus 6 g per serving) than the original version [191].

One concern about PBMAs is their potential to be consumed similarly to the traditional way in which hamburgers are consumed—with a refined bun and perhaps fries or chips [159]. Although this concern is understandable, in addition to patties, PBMAs are now available in many other forms. For example, products that emulate steak, chicken, and seafood are either already available or soon expected. Thus, the way PBMAs can be consumed is much broader than was initially the case.

## Hybrid Meat Products

Hybrid meat products, which refers to the combination of animal and plant protein, may be an especially appealing and efficacious approach to decreasing meat intake, as research shows that to create an effective dietary change, new practices should not diverge too much from consumers’ previous behavior [[192], [193], [194]]. These products preserve the animal flavor while reducing the amount of meat in the formulation. Such products offer convenience and a more characteristic taste to flexitarian consumers [195,196]. The purpose of hybrid meat products is to substitute a portion of the meat with a more sustainable source, whereas plant-based ingredients in meat products are often added for their functionality, such as extenders, fillers, and binders [84,197]. If hybrid meat products increase in popularity, soy protein ingredients can contribute to their success, as manufacturers have considerable experience using these combinations [198,199]. This is also likely the case for other legume proteins.

## Conclusion and Perspective on the Protein Intake Transition

Evidence indicates that PBMAs represent a convenient, nutritious, and sustainable way to lower the animal to plant protein intake ratio in developed countries. The occasional replacement of meat, such as beef, with a PBMA is unlikely to result in nutrient intake being compromised, especially if the PBMAs are fortified with shortfall micronutrients. A conservative estimate indicates that replacing just 4 servings per week of meat with 4 servings of soy-based PBMAs is sufficient to lower the animal to plant protein intake ratio from about 2.1:1 to 1.2:1, which is a reasonable goal given the glacial pace at which changes in dietary habits typically occur. As new PBMAs are developed, additional research on these products will be needed to help further understand their role in a healthy diet.

Future iterations of PBMAs should reflect a continued emphasis on improving nutrient content while maintaining the orosensory properties that make them effective options for individuals moving away from an animal-based to a more plant-predominant diet. In some sense, the comparison between the health attributes of legumes and PBMAs is irrelevant if in fact, legume intake is unlikely to substantially increase, although continued efforts aimed at increasing legume intake should be undertaken.

Health professionals need to be well informed about PBMAs so they can competently advise their patients and clients about the role these foods can play in the diet. Both health professionals and consumers need to recognize that the nutrient content of PBMAs varies markedly. Such variation also exists for plant-based milks; for example, some are low in protein and not all are fortified with key nutrients found in cow’s milk, such as calcium and vitamin D [200]. Recently, Klapp et al. [46] recommended that dietary guidelines “should differentiate between plant-based alternatives that can be consumed frequently and those that should be consumed in moderation or merely for enjoyment.”

In a recently published textbook on vegetarian diets for dietitians, intake recommendations for vegans included legumes at least 3 times daily [201]. Although both pulses/dry beans and soybeans are legumes, because of the high fat content of the latter and because of the many forms in which soybeans are consumed, soyfoods can be viewed as a separate category from pulses. Given their high fat content, peanuts (and peanut butter) can also be viewed similarly to soy [108]. The pulses/dry beans and soy/peanut categories can be part of legume-based PBMAs. Thus, there are 3 distinct ways to incorporate legumes into the diet, i.e., dry beans/pulses, peanuts and foods derived from soybeans, and PBMAs.

In the interest of diet/nutritional diversity and convenience, consumers may consider choosing foods from each of these 3 legume categories because doing so may enhance adherence—by increasing variety—to plant-predominant diets, and it may facilitate sufficient nutrient intake. On a per serving basis, many PBMAs are considerably higher in calories than pulses [140] and traditional soyfoods such as tofu (Table 3), so when caloric intake is restricted, this difference needs to be appreciated. Meeting the legume intake recommendation by consuming foods from the 3 legume-based food categories needs to be individualized based on health and personal factors including cultural considerations, economic status, and overall lifestyle.

In conclusion, PBMAs provide a convenient option for omnivores to transition to a diet that has a lower animal to plant protein ratio. As more such products enter the market, and their cost decreases as is expected, PBMAs are likely to become increasingly mainstream. For adherents of plant-predominant diets, well-designed, nutrient-dense formulations of PBMAs should be able to provide shortfall nutrients, and for many individuals, they will likely enhance compliance to their diet by increasing the variety of protein-rich plant foods available. Finally, whereas a deeper redesign of the diet to include more traditional, minimally processed plant protein sources that have proven nutritional/health benefits should remain a primary goal, PBMAs are useful and indeed practically indispensable to facilitate and maintain the transition toward a more plant-predominant diet for a large part of the population and are apt to make continued adherence to these diets easier.
